# MRI-Based Radiotherapy Planning to Reduce Rectal Dose in Excess of Tolerance

**DOI:** 10.1155/2022/7930744

**Published:** 2022-02-03

**Authors:** Daniel R. Schmidt, Mandar Bhagwat, Daniel I. Glazer, Ming-Hui Chen, Maryam Moteabbed, Elizabeth McMahon, Marian J. Loffredo, Clare M. Tempany, Anthony V. D'Amico

**Affiliations:** ^1^Department of Radiation Oncology, Beth Israel Deaconess Medical Center, 330 Brookline Ave, Boston, MA 02215, USA; ^2^Harvard Medical School, 25 Shattuck St, Boston, MA 02115, USA; ^3^Department of Radiation Oncology, 75 Francis St, Brigham and Women's Hospital, Boston, MA 02115, USA; ^4^Dana Farber Cancer Institute, 450 Brookline Ave, Boston, MA 02215, USA; ^5^Department of Radiation Oncology, Massachusetts General Hospital, 55 Fruit Street, Boston, MA 02114, USA; ^6^Department of Radiology, 75 Francis St, Brigham and Women's Hospital, Boston, MA 02115, USA; ^7^Department of Statistics, University of Connecticut, Philip E. Austin Bldg, 3rd floor, Storrs, CT 06269, USA

## Abstract

**Materials and Methods:**

This prospective single-arm study enrolled 15 men treated with IG-IMRT for localized prostate cancer. All participants received a dedicated 3 Tesla MRI examination of the prostate in addition to a pelvic CT examination for treatment planning. Two volumetric modulated arc therapy (VMAT) plans with a prescription dose of 79.2 Gy were designed using identical constraints based on CT- and MRI-defined consensus volumes. The volume of rectum exposed to 70 Gy or more was compared using the Wilcoxon paired signed rank test.

**Results:**

For CT-based treatment plans, the median volume of rectum receiving 70 Gy or more was 9.3 cubic centimeters (cc) (IQR 7.0 to 10.2) compared with 4.9 cc (IQR 4.1 to 7.8) for MRI-based plans. This resulted in a median volume reduction of 2.1 cc (IQR 0.5 to 5.3, *P* < .001).

**Conclusions:**

Using MRI to plan prostate IG-IMRT to a dose of 79.2 Gy reduces the volume of rectum receiving radiation dose in excess of tolerance (70 Gy or more) and should be considered in men who are at high risk for late rectal toxicity and are not good candidates for other rectal sparing techniques such as hydrogel spacer. This trial is registered with NCT02470910.

## 1. Introduction

Curative treatment of prostate cancer with radiation therapy can cause long-term urinary, bowel, and sexual side effects that may significantly impact quality of life [1–4]. Some of the more debilitating side effects are the result of rectal toxicity, including frequent and/or loose bowel movements, rectal urgency, tenesmus, fecal leakage, and rectal bleeding.

Technical innovations such as intensity modulated radiation therapy (IMRT), image-guidance with intraprostatic fiducial markers, and hydrogel rectal spacers have reduced the incidence of rectal toxicity [5–8]. However, not all patients are good candidates for a hydrogel spacer implant and optimal geometric insertion of the spacer occurs in only about two-thirds of cases. Furthermore, dose-escalated radiation therapy, which has been shown to decrease the incidence of biochemical recurrence and distant metastasis [9–11], has also made it more challenging to meet the validated dose constraints to the rectum in order to minimize the risk of late rectal toxicity [12].

It is well established that the probability of developing clinically significant rectal toxicity is related to the volume of rectum exposed to radiation [13–15]. It has also been recognized that clinical factors increase the risk of rectal toxicity including older age, use of anticoagulants, inflammatory bowel disease, diabetes, smoking, vascular disease, and history of abdominal surgery or prior pelvic radiation [16, 17]. Several mathematical models have been developed to predict the probability of rectal toxicity based on the rectal dose-volume histogram (DVH) and clinical factors [17, 18]. These models were developed using data from older studies and have not yet been validated using current prostate radiotherapy techniques and doses. In contrast, the rectal wall receiving high-dose radiation (≥70 Gy) is a validated predictor of chronic rectal toxicity in patients treated with IG-IMRT and prescription doses that exceed 78Gy [6, 13, 19–22].

While CT is the most widely used imaging modality for prostate radiotherapy planning, due to improved visualization of soft tissue anatomy, it is possible to more accurately define the prostate volume on MRI. The prostate volume has consistently been shown to be smaller on MRI compared to CT [23–25]. Furthermore, it has been shown when MRI is used to define the prostate volume, rectal dose is reduced for 3D-conformal radiation therapy (3D-CRT) plans prescribed to a dose of 78 Gy [26]. Image-guidance using intraprostatic fiducial markers and intensity-modulated radiation techniques such as volumetric modulated arc therapy (VMAT) allow more conformal doses to be delivered with smaller planning tumor volume (PTV) margins and have made it possible to increase the prescription dose while maintaining low dose to the rectum. Whether MRI-based radiation therapy planning further improves rectal dose is not known.

A downside of MRI is that it lacks information on tissue density (needed to calculate absorbed dose); thus, additional steps are need for dosimetry, which makes MRI-based planning less efficient than CT-based planning. While multiple studies have shown that MRI-based radiotherapy planning is feasible [27–30], concerns about increased resource utilization and inconvenience have been the major barriers to its widespread use. We propose that the additional resources needed for MRI-based radiotherapy planning are warranted if there is potential for clinical benefit.

To address these issues, we sought to determine whether MRI as compared to CT-based planning could reduce the volume of rectum receiving 70 Gy or more when the prostate is treated with 79.2 Gy using VMAT. To simulate a real-life clinical workflow and minimize bias, we conducted a prospective investigation in which study participants consented to a dedicated research MRI that was performed at the time of radiation therapy planning. As an important first step to documenting potential clinical benefit, we found that the rectal V70, a validated metric that predicts the risk of late rectal toxicity, was significantly decreased with MRI-based prostate radiation treatment planning. This finding was initially reported as an abstract at the 2020 American Society of Clinical Oncology Annual Meeting [31]. Herein, we present a full analysis of the data, which show that both rectal and bladder volume exposed to high dose radiation are significantly decreased with MRI-based planning, and that the greatest uncertainly with CT-based prostate volume delineation is at the prostate apex where clinically significant disease is frequently found.

## 2. Materials and Methods

### 2.1. Patient Selection

Fifteen men with newly diagnosed nonmetastatic prostate adenocarcinoma were enrolled on an interventional research protocol (DFCI 14-585) approved by the IRB at the Dana-Farber Cancer Institute. Inclusion criteria were stage I-III prostate cancer, with no prior curative local treatment, and maximum 90 days of androgen deprivation therapy prior to registration. The study was registered at ClinicalTrials.gov (NCT02470910) prior to enrollment.

### 2.2. Interventions

In addition to the standard-of-care CT examination, participants underwent an unenhanced MRI examination of the prostate without an endorectal coil at the time of treatment planning. All examinations were performed on a 3.0 Tesla MRI device (Signa HDxt 3T, GE Healthcare) and utilized a pelvic-phased array coil. MRI and CT examinations were performed on the same day and at least 1 week after the placement of intraprostatic fiducial markers. To allow reproducible positioning of the prostate on both examinations, participants were instructed to maintain a full bladder and empty rectum. T1-weighted images were generated from spoiled gradient echo (SPGR) sequences, and T2-weighted images were generated from fast relaxation fast spin echo sequences (FRFSE). Slice thickness for all pulse sequences was 3 mm. After acquisition, images were uploaded to a research picture archiving and communication system (PACS) for subsequent analysis.

### 2.3. Definition of Target Volumes and Organs at Risk (OARs)

Consensus volumes of prostate, rectum, and bladder were defined on the research MRI by a subspecialty trained abdominal radiologist along with a radiation oncologist and on the planning CT scan by two radiation oncologists without prior review of the MRI exam. The prostate volume on CT was defined according to published guidelines [32]. The prostate gland was defined as the clinical tumor volume (CTV) and additional 1 cm of the adjacent seminal vesicles (SV) as CTV + 1 cm SV. MRI volumes were transferred to the CT scan using rigid image registration based on the alignment of intraprostatic fiducial markers. In order to understand where differences in the MRI- and CT-defined prostate volumes existed, a quadrant-based analysis was performed. After fiducial-based image registration of MRI and CT, a centroid (volumetric center of gland) was defined, and the prostate was divided by quadrant (anterior-base, posterior-base, anterior-apex, and posterior apex).

### 2.4. Treatment Planning

A uniform planning tumor volume (PTV) expansion of 5 mm was used. 2-Arc volumetric modulated arc therapy (VMAT) plans were generated using Eclipse™ Treatment Planning System, version 11.0 (Varian Medical Systems, Palo Alto, CA) for a dose of 79.2 Gy to be delivered in 44 fractions. Each arc had an angular range of 340°. The start and stop angles, 170° and 190°, were chosen to minimize entrance dose through the rectum. Separate plans were created for CT- and MR-defined CT contours. Identical constraints were imposed on the PTV and OARs for each contour and patient plan during optimization to prevent planning bias. The plans were normalized such that 99% of the PTV was covered by the prescription dose of 79.2 Gy. The absolute volume of rectum and bladder in cc receiving 70 Gy or more (V70), 75 Gy or more (V75), and 80 Gy or more (V80) was calculated from the dose-volume histogram (DVH).

### 2.5. Tumor Control Probability (TCP) and Normal Tissue Complication Probability (NTCP) Modeling

Mathematical models that incorporate a dose-volume variable and empirically determined radiobiologic constants have been used to estimate the TCP and NTCP [33–36]. TCP and NTCP calculations were performed using the equivalent uniform dose (EUD)-based model [37]. For tumors, the EUD is the single dose (in Gy) applied uniformly to the tumor that that kills the same number of clonogens as a given plan with an inhomogeneous dose distribution [36]. For normal tissues, the EUD represents the uniform dose that leads to the same probability of injury as a given plan with an inhomogeneous dose distribution.

The DVH for the rectum and prostate gland, hereafter referred to as the clinical tumor volume (CTV), were extracted from the treatment planning system for each CT-based and MRI-based plan. To estimate coverage of the “true” prostate by the plan that was optimized to the CT-defined prostate (the CT-based plan), the MR-CTV (considered the “true” prostate volume) was mapped onto the CT-based plan, and the resulting DVH was extracted. The dose volume histograms were converted to EUDs using the equation:

(1) $$\begin{array}{c} \text{EUD}={\left(\sum_{i}v_{i}D_{i}^{a}\right)}^{\left(1/a\right)}, \end{array}$$

where *v*_*i*_ is the fractional organ volume receiving a dose *D*_*i*_ and *a* is a tissue-specific parameter that describes the volume effect. To minimize cold spots within the tumor the parameter *a* was set equal to −10 for the CTV. For rectum, a serial organ, the parameter *a* was set to 8 [37].

The TCP was calculated using the equation:

(2) $$\begin{array}{c} \text{TCP}=\frac{1}{1+{\left({\text{TCD}}_{50}/\text{EUD}\right)}^{4\gamma 50}}, \end{array}$$

where TCD_50_ (the dose at which the probability of controlling the tumor is equal to 50 percent) was set to 72 Gy for the PTV for an endpoint of 5-year freedom from recurrence, and the *γ*50 parameter (which represents the slope of the dose response curve around a TCP of 50%) was set to 5 based prior estimates of these factors for intermediate- to high-risk prostate cancer [38].

The NTCP was calculated using the equation:

(3) $$\begin{array}{c} \text{NTCP}=\frac{1}{1+{\left({\text{TD}}_{50}/\text{EUD}\right)}^{4\gamma 50}}, \end{array}$$

where TD_50_ (the dose at which the probability of toxicity is equal to 50 percent) was set to 76.9 Gy for late rectal toxicity (late rectal bleeding) ≥ grade 2 based on the literature [13]. Two different values for the *γ*50 parameter were evaluated based on prior reports [12, 39].

Data analysis was performed using an inhouse script (Root5.34/36, https://root.cern, on MacOS10.12.6).

### 2.6. Statistical Methods

Descriptive statistics were used to characterize the clinical characteristics of the patient population at enrollment. For the dosimetric comparison, normality could not be demonstrated in all datasets; therefore, we used the Wilcoxon paired signed rank test [40] to compare rectal and bladder V70, V75, and V80 for MRI- versus CT-based plans, which were the primary and secondary study endpoints, respectively. TCP and NTCP for MRI- versus CT-based plans were compared using paired Student's *t*-test or Wilcoxon paired signed rank test. Prostate volumes on CT and MRI were compared using Pearson correlation coefficient and paired Student's *t*-test. Rectum volumes on CT and MRI were compared using Spearman correlation coefficient and Wilcoxon paired signed rank test. The difference between the CT- and MRI-defined planning tumor volume (PTV) by quadrant was compared using paired Student's *t*-test or Wilcoxon paired signed rank test. SAS version 9.4 and Prism version 7.0a were used for statisitcal analysis. All *P* values are from two-sided tests. Additional details of statistical comparisons are included in the supplementary data file.

## 3. Results

### 3.1. Clinical Characteristics

The median age was 70 years (range 56–84), median PSA was 7.3 ng/mL (range 3.2–22.1), and median prostate volume was 40 mL (range 25–65) by transrectal ultrasound. Sixty percent (*n* = 9) had intermediate risk, and 40 percent (*n* = 6) had high risk by NCCN criteria. The majority were either clinical stage T1c (*n* = 7) or T2 (*n* = 6). Two men had extracapsular extension (T3a). None of the participants had seminal vesicle invasion (T3b), rectal or bladder involvement (T4), or lymph node metastasis (N1).

### 3.2. Rectal Dose on CT- versus MRI-Based Radiotherapy Plans

**Figure 1:**
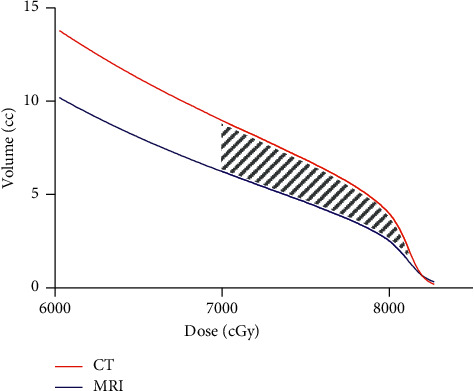
Rectum dose-volume histogram for CT- versus MRI-based planning. The volume of rectum at each dose level in 8 cGy intervals was averaged over 15 trial participants and is shown as a single dose-volume histogram for CT- versus MRI-based radiotherapy plans. Hatched area shows volume reduction of expected clinical benefit.

The volume of rectum receiving high dose radiotherapy was significantly lower when MRI-based radiation therapy planning was used compared to CT-based planning (Figure 1 and Table 1). Specifically, the ∆V70, ∆V75, and ∆V80 (defined as the difference between rectal volume on CT versus MRI receiving 70 Gy or more, 75 Gy or more, and 80 Gy or more, respectively) was statistically significant (Table 1). The volume reduction was also significant when the proximal 1 cm of the seminal vesicles was included in the target volume (Table 1).

### 3.3. Bladder Dose on CT- versus MRI-Based Radiotherapy Plans

MRI-based planning also resulted in a smaller volume of bladder exposed to high-dose radiation (Table 2) that was statistically significant when radiation was prescribed only to the prostate. When the treatment volume was extended to include the proximal 1 cm of the seminal vesicles, the volume reduction was even more significant (Table 2).

### 3.4. Comparison of Prostate and Rectal Volumes on CT versus MRI

The mean rectum volume was similar on MRI and CT (60.2 versus 51.1 cc, *P* = 0.52) and correlated well between the two modalities for most but not all patients (Spearman *r* = 0.479, 95% CI −0.061 to 0.802, *P* = 0.07). In line with prior reports [23–25], the mean prostate volume was on average smaller on MRI than on CT (30.8 versus 46.0 cc, *P* < 0.001). Prostate volume also correlated well between the two modalities for each patient (Pearson *r* = 0.854, 95% CI 0.607 to 0.950, *P* < .001).

**Figure 2:**
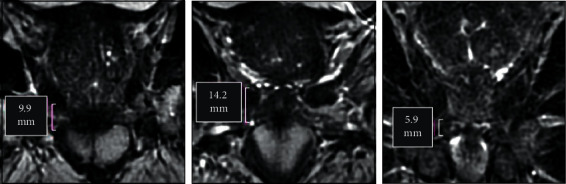
Anatomic variation at the prostate apex. Length of the urogenital diaphragm measured on MRI in the coronal plane is shown.

Although prostate volume on MRI is smaller than on CT, it is not a concentric volume reduction. We observed the greatest variance at the prostate apex where the interface between prostate and urogenital diaphragm is particularly difficult to define on CT. In most cases, the CT-defined volume overestimated the true volume of the prostate at the apex, where on average 30 percent of the CT volume extended outside the MRI volume; however, in 2 of 15 cases, MRI showed a small portion of the prostate apex outside the CT volume. In this series of 15 men, we found considerable variation in the height of the urogenital diaphragm that could only be accurately defined on MRI (Figure 2).

**Figure 3:**
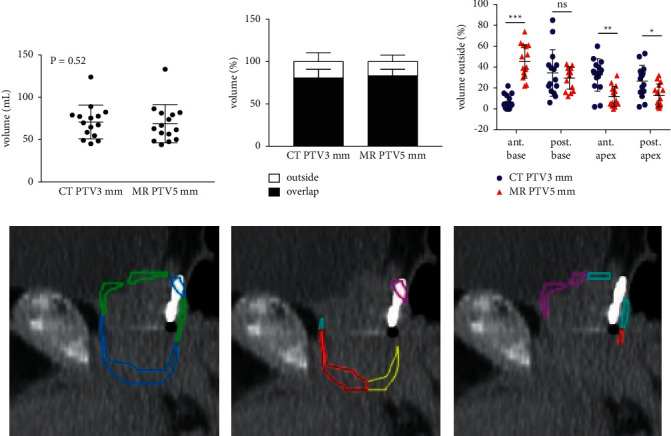
Discordance between CT and MR prostate contour by quadrant. Absolute volume (a) and percent overlap (b) of CT PTV3 mm and MR PTV5 mm are shown. (c) Percent volume by quadrant where the CT PTV3mm extends outside MR PTV5 mm is shown in blue, and percent volume by quadrant where the MR PTV5 mm extends outside the CT PTV3 mm is shown in red. Graphical representation of regions of discordance between CT PTV3 mm and MR PTV5 mm is shown in the panels. (d) Volume in green shows areas were the MR PTV5 mm extends outside the CT PTV3 mm, and volume in blue shows areas were the CT PTV3 mm extends outside the MR PTV5 mm. (e) Location (by quadrant) where CT PTV3 mm extends outside the MR PTV5 mm is shown. (f) Location (by quadrant) where MR PTV5 mm extends outside the CT PTV3 mm is shown. All panels show mean ± standard deviation. In panels A and C, individual values for all 15 subjects are shown. ^*∗*^*P* < 0.05, ^*∗∗*^*P* < 0.01, and ^*∗∗∗*^*P* < 0.001 by two-tailed paired Student's *t*-test or signed rank test.

To further illustrate that the prostate volume on MRI is not simply a concentric reduction of the CT-based volume, we decreased the CT PTV margin to 3 mm (CT PTV3 mm) and compared it to a standard 5 mm PTV expansion on the MRI-based volume (MR PTV 5 mm). When the CT PTV3mm is compared to MR PTV 5 mm, the absolute volume is similar (Figure 3(a)); however, 10–15% of the volumes do not overlap (Figure 3(b)). While the CT PTV3mm was still larger than the MR PTV5 mm at the apex, at the anterior base, it was significantly smaller, demonstrating that portions of the anterior base potentially could be missed if the CT PTV were reduced by 2 mm (Figures 3(c)–3(f)).

### 3.5. Probability Models to Compare Tumor Control and Rectal Complication Risk

**Figure 4:**
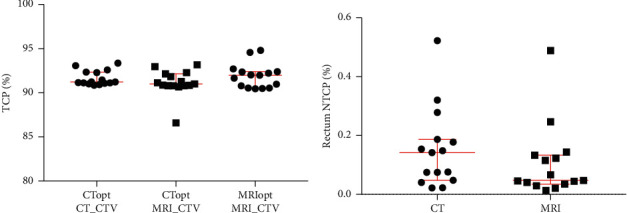
Comparison of TCP and NTCP for CT- versus MRI-based planning. The percent tumor control probability (a) and percent rectal complication probability (b) for CT-based and MRI-based radiotherapy planning is shown. None of the differences between groups were significant. See text for TCP group definitions.

The TCP of the CT-defined CTV was similar to the TCP of the MRI-defined CTV (mean ΔCT-MRI = −0.24423, standard deviation 1.23801, *P* = 0.4575), indicating that CT- and MRI-based plans performed similarly with regard to predicted tumor control for the CTV defined by CT and MRI respectively (CTopt CT-CTV and MRIopt MRI-CTV) (Figure 4(a)). Since the MRI-defined prostate more accurately represents the “true” prostate, particularly at the prostate apex, we also calculated the TCP of the MRI-defined CTV for the plan that was optimized to the CT-defined PTV (CTopt MRI-CTV). For all but one subject, the TCP of the MR-defined CTV was similar to TCP of the CT-defined CTV (median ΔCT-MRI = −0.8506, interquartile range 1.9873, *P* = 0.1688), indicating that, for most patients, the standard PTV expansion of 5 mm sufficiently covers differences between the CT-defined and MRI-defined prostate CTV (Figure 4(a)).

NTCP modeling, which takes into account the entire dose distribution, showed no statistically significant difference in predicted grade 2 or higher late rectal toxicity between MRI-based and CT-based radiotherapy planning (for dose prescribed to prostate only and *γ*_50_ = 8, the mean ΔCT-MRI = 0.0463, standard deviation 0.17078, *P* = 0.3114), but trended in favor of MRI-based planning (Figure 4(b) and supplemental data).

## 4. Discussion

Compared to CT-based planning, MRI-based IG-IMRT prostate radiotherapy planning, with a prescription dose of 79.2 Gy, results in a significantly lower volume of rectum receiving 70 Gy or more. We focused our analysis on the volume of rectum receiving high dose radiation because multiple studies have confirmed the volume of rectum exposed to ≥70 Gy is predictive of grade ≥2 late rectal toxicity (primarily rectal bleeding), while mixed results have been observed for more moderate doses (40–50 Gy) [6, 13, 19–22]. NTCP models have been used to estimate probability of late rectal toxicity by taking into account the entire dose distribution. Such dose-response models have been fitted to clinical and dosimetric data to derive radiobiologic parameters for rectal tolerance, yet their performance on different datasets has not always been consistent [39, 41, 42]. We did not find that MRI-based planning significantly improved the NTCP; however, differences in NTCP for MRI versus CT-based plans trended in favor of MRI-based planning, likely reflecting the benefits observed in the high dose range.

We found that the main reason for lower rectal dose was due to less contact between prostate and rectum at the posterior prostate apex due to significantly improved anatomic resolution of the prostate apex on MRI. Although the MRI-defined prostate volume is smaller than the CT-defined volume, we were interested to find that the size difference could not be explained by a concentric reduction in prostate size on MRI compared with CT. This means that the larger prostate size on CT cannot be viewed as an additional safety margin. Reducing the CT PTV by 2 mm results in an absolute volume that is similar to the MRI PTV; however, up to 15% of the MRI PTV extends outside reduced-volume CT PTV. Thus, simply reducing the CT PTV increases risk of missing tumor that is in the margin of error accounted for by a standard 5 mm PTV margin.

TCP modeling indicated that CT- and MRI-based plans performed similarly with regard to predicted tumor control. This was expected since both plans were optimized to provide similar coverage of the prostate gland as defined on the respective imaging modalities. Although this analysis confirms that the two forms of planning allowed for similar coverage of the CTV as defined by CT or MRI, it does not address the more important question of whether the CT plan adequately covers the “true prostate” volume. To address this, we also investigated TCP of the MR-CTV mapped onto the CT-based plan. For one patient, the TCP of the MR-CTV mapped onto the CT optimized plan was lower than expected, indicating that, for a small subset of men MRI-based planning may improve oncologic outcomes by preventing marginal misses that may occur by inadequate definition of the prostate, particularly at the apex where 40% of clinically significant cancers exist.

Although a bladder dose-volume correlation with urinary toxicity is less well defined, it is generally accepted that a greater volume of bladder in the high-dose radiation field is associated with greater incidence of acute and chronic toxicity. We also found that MRI-based planning resulted in a smaller volume of bladder exposed to high-dose radiation, particularly when the treatment target included the proximal seminal vesicles, a common practice for high-risk prostate cancer.

Our study has several limitations. Due to institutional practice at the time the study was conducted, low risk prostate cancer was primarily managed with active surveillance; thus, no men with low risk disease were enrolled on this study. Notwithstanding, the target volume and dose are the same for low and intermediate risk prostate cancer; therefore, it is reasonable to conclude that the findings of this study could also be applied to low risk prostate cancer. Secondly, for some intermediate- and high-risk prostate cancers, it is common practice to include a portion of the seminal vesicles in the target volume; however, there is no consensus on what length of seminal vesicles to treat. Significant reduction in rectum and bladder volume in the high dose radiation field was observed in this study when the target was defined as prostate only or prostate plus 1 cm of the proximal seminal vesicles. Finally, with regard to accuracy of prostate contours, the prostate volume on MRI was defined by a radiation oncologist and a subspecialty trained abdominal radiologist, while on CT, the volume was defined by consensus of two radiation oncologists with subspecialty in genitourinary cancers. It is not known whether CT-defined prostate volume could be more accurately defined by a radiologist; however, it is standard practice for prostate volume to be defined by the radiation oncologist, and care was taken on this study to adhere to published guidelines [32].

The clinical significance of the findings of this study is that MRI-based radiation therapy planning is a noninvasive tool that has the potential to significantly reduce the risk of chronic rectal toxicity and should be considered in men who are at high risk for this late side effect and for whom a rectal spacer (another approach that can reduce the volume of rectum receiving high-dose RT) is not possible or desired. This includes men with diabetes, vascular disease, coagulopathy, use of on antiplatelet drugs and anticoagulants, history of inflammatory bowel disease, history of abdominal surgery, and unfavorable anatomy, including prostatic hypertrophy and minimal fat between prostate and rectal wall.

MRI-based planning may also decrease the risk of urinary toxicity, particularly in cases where the proximal seminal vesicles are treated. Additionally, there exists the opportunity for improved cancer control due to more accurate definition of the prostate apex (where 40% of prostate cancer is known to exist) on MRI compared with CT imaging. Finally, with ongoing improvements in technology, MRI-based planning would also make it possible to focally increase dose to areas in the prostate with imaging characteristics indicative of aggressive or radioresistant disease.

The primary goal of this study was to show that utilizing MRI for prostate radiation therapy planning in a high-volume academic center has the potential to decrease toxicity based on a validated metric (volume of rectum exposed to 70 Gy or more). All men on our single-arm study received treatment per the current standard of care (CT-based radiotherapy planning). A prospective comparison of CT- versus MRI-planned prostate radiotherapy with regard to cancer control and patient reported outcomes is warranted.

## 5. Conclusions

In this single-arm prospective study, we found that prostate volume on CT is primarily overestimated at the prostate apex. We demonstrate that MRI-planned prostate radiotherapy significantly reduces rectal V70, a validated metric shown to correlate with rectal toxicity. We propose that MRI-based planning should be considered for men at high risk for late rectal toxicity and for whom other rectal sparing techniques, such as hydrogel spacer, are not available or desired.

## Figures and Tables

**Table 1:** Rectal volume exposed to high dose radiation.

	CT	MRI	Δ(CT − MRI)	*P* value
*Rx to prostate only*				
Median cc (IQR)				
V70	9.3 (7.0, 10.2)	4.9 (4.1, 7.8)	2.1 (0.5, 5.3)	<0.001
V75	7.2 (5.6, 8.0)	3.8 (3.1, 5.7)	1.6 (0.7, 4.1)	<0.001
V80	4.0 (3.0, 4.8)	2.1 (1.5, 3.2)	0.9 (0.3, 2.6)	<0.001

*Rx to prostate* *+* *1 cm SVs*				
Median cc (IQR)				
V70	10.9 (9.4, 11.6)	6.7 (5.4, 9.3)	2.7 (0.4, 5.2)	0.007
V75	8.5 (7.0, 9.1)	4.7 (4.1, 7.0)	1.7 (−0.1, 4.3)	0.005
V80	4.6 (3.7, 5.8)	2.7 (2.2, 4.3)	0.9 (0.2, 3.0)	0.003

V70, volume of organ that receives 70 Gy or higher; V75, volume of organ that receives 75 Gy or higher; V80, volume of organ that receives 80 Gy or higher; IQR, interquartile range; cc, cubic centimeters.

**Table 2:** Bladder volume exposed to high dose radiation.

	CT	MRI	Δ(CT − MRI)	*P* value
*Rx to prostate only*				
Median cc (IQR)				
V70	12.7 (10.8, 15.0)	9.6 (8.2, 12.9)	3.0 (−0.8, 4.7)	0.006
V75	10.2 (9.0, 12.2)	7.8 (6.6, 10.4)	2.3 (−0.9, 3.7)	0.008
V80	7.4 (6.4, 8.8)	5.6 (4.5, 7.2)	1.4 (−0.7, 3.2)	0.011

*Rx to prostate* *+* *1 cm SVs*				
Median cc (IQR)				
V70	16.5 (14.8, 17.7)	10.6 (9.4, 13.7)	5.1 (2.9, 6.8)	<0.001
V75	13.2 (11.9, 14.5)	8.7 (7.6, 11.2)	4.0 (2.2, 5.9)	<0.001
V80	9.1 (8.4, 10.9)	6.5 (5.6, 8.1)	2.7 (1.3, 4.4)	<0.001

V70, volume of organ that receives 70 Gy or higher; V75, volume of organ that receives 75 Gy or higher; V80, volume of organ that receives 80 Gy or higher; IQR, interquartile range; cc, cubic centimeters.

## Data Availability

Raw data supporting the conclusions of this study are freely available upon request.

## Supplementary Materials

Supplementary Materials All supplementary data are provided as a single PDF file. The data are separated into sheets that correspond to the main subsections, tables, and figures in manuscript. A brief description of the data in each sheet is provided below: Sheet 1 (data for Table 1): raw values and normality test for data shown in Table 1. Sheet 2 (data for Table 2): raw values and normality test for data shown in Table 2. Sheet 3 (prostate volume): raw values, statistical analysis, and graph comparing prostate volumes on CT versus MRI. Sheet 4 (rectum volume): raw values, statistical analysis, and graph comparing rectum volumes on CT versus MRI. Sheet 5 (data for Figure 3(a)): raw values and statistical analysis for data shown in Figure 3(a). Sheet 6 (data for Figure 3(b)): raw values and statistical analysis for data shown in Figure 3(b). Sheet 7 (data for Figure 3(c)): raw values and statistical analysis for data shown in Figure 3(c). Sheet 8 (TCP): raw values and statistical analysis comparing TCP for CT versus MRI-based plans. Sheet 9 (NTCP): raw values and statistical analysis comparing NTCP for CT versus MRI-based plans. Sheet 10 (all raw volume data): raw values of volume and spatial comparison for CTV and PTV on CT and MRI that were used to generate the data in Figures 3(a)–3(c).
