# Supplementary material for: MRI-Based Radiotherapy Planning to Reduce Rectal Dose in Excess of Tolerance
Source: Prostate Cancer. 2022 Feb 3;2022:7930744. doi: 10.1155/2022/7930744 (PMC8831048; doi:10.1155/2022/7930744)
Supplement: Supplementary Materials — All supplementary data are provided as a single PDF file. The data are separated into sheets that correspond to the main subsections, tables, and figures in manuscript. A brief description of the data in each sheet is provided below: Sheet 1 (data for Table 1): raw values and normality test for data shown in Table 1. Sheet 2 (data for Table 2): raw values and normality test for data shown in Table 2. Sheet 3 (prostate volume): raw values, statistical analysis, and graph comparing prostate volumes on CT versus MRI. Sheet 4 (rectum volume): raw values, statistical analysis, and graph comparing rectum volumes on CT versus MRI. Sheet 5 (data for Figure 3(a)): raw values and statistical analysis for data shown in Figure 3(a). Sheet 6 (data for Figure 3(b)): raw values and statistical analysis for data shown in Figure 3(b). Sheet 7 (data for Figure 3(c)): raw values and statistical analysis for data shown in Figure 3(c). Sheet 8 (TCP): raw values and statistical analysis comparing TCP for CT versus MRI-based plans. Sheet 9 (NTCP): raw values and statistical analysis comparing NTCP for CT versus MRI-based plans. Sheet 10 (all raw volume data): raw values of volume and spatial comparison for CTV and PTV on CT and MRI that were used to generate the data in Figures 3(a)–3(c). [file 7930744.f1.pdf]

This section shows the rectal V70, V75, V80 (planning to prostate only)

	CT V70 (cc)	MR V70 (cc)	deltaV70	CT V75 (cc)	MR V75 (cc)	deltaV75	CT V80 (cc)	MR V80 (cc)	deltaV80
patient 1	8.19	5.64	2.55	5.79	4.24	1.55	2.49	1.99	0.50
patient 2	5.81	4.11	1.70	4.49	3.23	1.26	2.75	2.06	0.69
patient 3	5.13	2.99	2.14	3.74	2.06	1.68	2.10	1.16	0.94
patient 4	6.81	6.27	0.54	5.35	4.49	0.86	3.28	2.25	1.03
patient 5	12.33	4.92	7.41	9.77	3.75	6.02	6.35	2.41	3.94
patient 6	10.24	4.94	5.30	7.84	3.77	4.07	4.58	2.27	2.31
patient 7	10.09	9.79	0.30	8.10	7.43	0.67	5.05	4.72	0.33
patient 8	9.37	8.69	0.68	7.15	6.76	0.39	4.10	4.07	0.03
patient 9	7.31	3.55	3.76	5.84	2.46	3.38	3.95	1.17	2.78
patient 10	6.98	6.76	0.22	4.69	4.61	0.08	1.53	1.56	-0.03
patient 11	8.37	4.50	3.87	6.42	3.25	3.17	3.78	1.39	2.39
patient 12	14.24	13.38	0.86	10.72	9.44	1.28	5.41	4.89	0.52
patient 13	10.20	3.92	6.28	8.17	2.93	5.24	4.99	1.68	3.31
patient 14	9.29	9.75	-0.46	7.37	7.47	-0.10	4.59	4.30	0.29
patient 15	10.09	4.25	5.84	7.59	2.90	4.69	3.70	1.09	2.61

normality test: Shapiro-Wilk test

P value			0.17			0.13			0.06

significance test: Wilcoxon paired signed rank test
see table 1 for estimate, confidence interval, and P value

This section shows the rectal V70, V75, V80 (planning to prostate + 1 cm SV)

	CT V70 (cc)	MR V70 (cc)	deltaV70	CT V75 (cc)	MR V75 (cc)	deltaV70	CT V80 (cc)	MR V80 (cc)	deltaV70
patient 1	10.09	6.73	3.36	6.99	4.72	2.27	2.97	1.98	0.99
patient 2	6.74	4.46	2.28	5.20	3.49	1.71	3.11	2.23	0.88
patient 3	6.50	4.42	2.08	4.53	3.28	1.25	2.27	1.90	0.37
patient 4	7.88	8.32	-0.44	6.39	6.17	0.22	4.15	3.41	0.74
patient 5	14.60	5.53	9.07	11.34	4.26	7.08	7.01	2.70	4.31
patient 6	13.26	6.79	6.47	10.45	5.39	5.06	6.87	3.73	3.14
patient 7	11.71	12.97	-1.26	9.43	9.77	-0.34	5.97	5.98	-0.01
patient 8	10.44	10.19	0.25	7.80	7.92	-0.12	4.60	5.15	-0.55
patient 9	8.73	4.35	4.38	6.94	3.07	3.87	4.55	1.59	2.96
patient 10	10.23	7.54	2.69	7.01	5.55	1.46	3.31	2.70	0.61
patient 11	10.99	6.60	4.39	8.82	4.72	4.10	5.72	2.64	3.08
patient 12	15.56	17.27	-1.71	11.80	12.52	-0.72	6.15	6.79	-0.64
patient 13	10.86	5.21	5.65	8.68	4.00	4.68	5.14	2.46	2.68
patient 14	10.94	11.39	-0.45	8.54	8.82	-0.28	5.12	4.92	0.20
patient 15	11.40	6.20	5.20	8.65	4.40	4.25	4.64	2.15	2.49

normality test: Shapiro-Wilk test

P value			0.76			0.25			0.18

significance test: Wilcoxon paired signed rank test
see table 1 for estimate, confidence interval, and P value

This section shows the bladder V70, V75, V80 (planning to prostate only)

	CT V70 (cc)	MR V70 (cc)	deltaV70	CT V75 (cc)	MR V75 (cc)	deltaV75	CT V80 (cc)	MR V80 (cc)	deltaV80
patient 1	8.50	9.25	-0.75	6.55	7.50	-0.95	4.09	5.24	-1.15
patient 2	11.26	7.38	3.88	9.28	6.05	3.23	6.92	4.51	2.41
patient 3	4.55	2.97	1.58	3.52	2.41	1.11	2.43	1.84	0.59
patient 4	10.43	6.83	3.60	8.78	5.50	3.28	6.87	3.87	3.00
patient 5	11.82	13.65	-1.83	9.44	10.89	-1.45	6.13	7.09	-0.96
patient 6	11.92	9.55	2.37	9.51	7.76	1.75	6.66	5.56	1.10
patient 7	26.03	16.40	9.63	20.54	13.42	7.12	14.90	10.10	4.80
patient 8	15.26	15.33	-0.07	12.56	12.65	-0.09	9.42	9.41	0.01
patient 9	12.66	7.94	4.72	10.20	6.49	3.71	7.39	4.53	2.86
patient 10	9.66	10.59	-0.93	7.45	8.50	-1.05	4.83	5.87	-1.04
patient 11	15.86	8.55	7.31	13.06	6.63	6.43	9.77	4.43	5.34
patient 12	14.18	11.21	2.97	11.43	9.11	2.32	7.66	6.29	1.37
patient 13	18.07	12.15	5.92	15.09	9.95	5.14	10.74	7.40	3.34
patient 14	12.66	8.74	3.92	10.50	7.08	3.42	7.90	4.68	3.22
patient 15	14.73	15.76	-1.03	11.92	12.84	-0.92	8.09	8.78	-0.69
normality test: Shapiro-Wilk test									
P value			0.57			0.34			0.30

significance test: Wilcoxon paired signed rank test
see table 2 for estimate, confidence interval, and P value

This section shows the bladder V70, V75, V80 (planning to prostate + 1 cm SV)

	CT V70 (cc)	MR V70 (cc)	deltaV70	CT V75 (cc)	MR V75 (cc)	deltaV70	CT V80 (cc)	MR V80 (cc)	deltaV70
patient 1	10.80	8.44	2.36	8.37	6.78	1.59	5.27	4.64	0.63
patient 2	14.89	10.42	4.47	12.46	8.66	3.80	9.35	6.49	2.86
patient 3	7.71	4.81	2.90	6.06	3.76	2.30	4.03	2.69	1.34
patient 4	10.62	7.69	2.93	8.90	6.24	2.66	6.98	4.54	2.44
patient 5	16.50	13.83	2.67	13.19	11.00	2.19	9.00	7.19	1.81
patient 6	14.68	11.61	3.07	11.63	9.44	2.19	8.12	7.03	1.09
patient 7	28.34	17.20	11.14	22.88	14.10	8.78	17.07	10.63	6.44
patient 8	20.39	15.33	5.06	17.16	12.84	4.32	12.76	10.05	2.71
patient 9	14.90	9.02	5.88	12.09	7.52	4.57	8.80	5.69	3.11
patient 10	17.44	10.62	6.82	13.41	8.54	4.87	9.14	5.92	3.22
patient 11	17.94	9.85	8.09	14.62	7.76	6.86	10.86	5.47	5.39
patient 12	16.60	11.46	5.14	13.30	9.31	3.99	8.71	6.55	2.16
patient 13	20.89	13.48	7.41	17.62	11.35	6.27	13.32	8.93	4.39
patient 14	17.12	10.28	6.84	14.32	8.44	5.88	10.86	5.97	4.89
patient 15	16.27	18.07	-1.80	13.19	15.05	-1.86	9.13	11.02	-1.89
normality test: Shapiro-Wilk test									
P value			0.82			0.91			0.95

significance test: Wilcoxon paired signed rank test
see table 2 for estimate, confidence interval, and P value

This section shows the prostate volumes (in cc) on CT and MRI

	CT prostate	MR prostate
patient 1	37.0	23.9
patient 2	29.9	28.7
patient 3	41.3	38.4
patient 4	59.6	37.4
patient 5	50.5	33.5
patient 6	31.0	19.0
patient 7	86.1	71.8
patient 8	51.5	38.0
patient 9	32.8	18.6
patient 10	53.9	40.2
patient 11	27.1	20.1
patient 12	50.9	27.1
patient 13	48.9	26.2
patient 14	45.2	15.6
patient 15	43.9	23.5
mean	46.0	30.8
standard deviation	14.8	13.9

normality test:	Shapiro-Wilk test
P value	0.78
significance test:	two-tailed paired student's t-test
P value	<0.0001
correlation analysis:	Pearson r
r	0.8539
95% confidence inte	0.6072 to 0.9504
R squared	0.7291
P value (two-tailed)	<0.0001

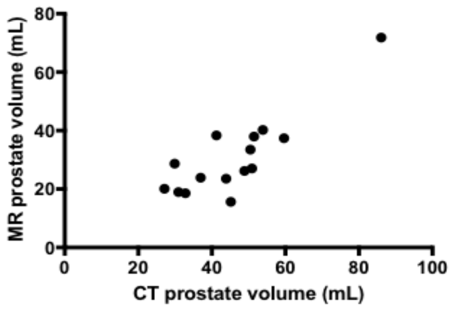

This section shows the rectal volumes (in cc) on CT and MRI

	CT rectum	MR rectum
patient 1	57.5	40.7
patient 2	52.7	63.1
patient 3	42.8	86.6
patient 4	44.6	41.3
patient 5	72.1	51.3
patient 6	28.0	24.6
patient 7	49.3	69.0
patient 8	77.9	92.9
patient 9	52.8	38.2
patient 10	88.4	184.7
patient 11	28.5	23.4
patient 12	43.8	47.9
patient 13	44.6	41.5
patient 14	35.7	61.9
patient 15	48.4	36.3
mean	51.1	60.2
standard deviation	17.1	40

normality test:	Shapiro-Wilk Test
P value	0.005
significance test:	Wilcoxon paired signed rank test
P value	0.52
correlation analysis:	Spearman r
r	0.4786
95% confidence inte	-0.06131 to 0.8018
P value (two-tailed)	0.0735

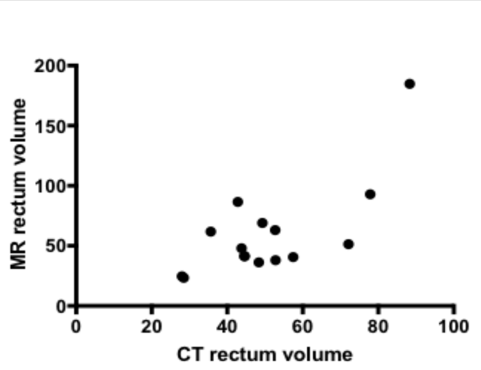

This section shows the volumes of the CT PTV3mm and the MR PTV5mm

ID	CT PTV3mm	MR PTV5mm	delta PTV
1	58.6	57.88	0.72
2	48.34	66.81	-18.47
3	64.73	81.34	-16.61
4	89.02	79.29	9.73
5	77.68	73.85	3.83
6	49.15	47.48	1.67
7	123.95	133.12	-9.17
8	78.95	81.94	-2.99
9	54.61	48.3	6.31
10	82.44	86.41	-3.97
11	45.06	50.17	-5.11
12	77.33	62.93	14.4
13	75.49	61.65	13.84
14	69.74	43.96	25.78
15	67.31	56.51	10.8
mean	70.83	68.78	2.05
standard deviation	19.94	22.54	12.02

normality test:	Shapiro-Wilk test
P value	0.99
significance test:	two-tailed paired student's t-test
P value	0.52

This section shows the MR PTV5mm overlap with CT PTV3mm

ID	%overlap	%outside
1	85%	15%
2	67%	33%
3	68%	32%
4	75%	25%
5	86%	14%
6	89%	11%
7	84%	16%
8	86%	14%
9	88%	12%
10	84%	16%
11	81%	19%
12	95%	5%
13	88%	12%
14	86%	14%
15	88%	12%
mean	83%	17%
standard deviation	7.69%	7.69%

This section shows the CT PTV3mm overlap with MR PTV5mm

ID	%overlap	%outside
1	84%	16%
2	92%	8%
3	86%	14%
4	67%	33%
5	82%	18%
6	86%	14%
7	90%	10%
8	89%	11%
9	77%	23%
10	88%	12%
11	90%	10%
12	77%	23%
13	72%	28%
14	54%	46%
15	74%	26%
mean	81%	19%
standard deviation	10.56%	10.56%

This section shows the MR PTV5mm extension outside CT PTV3mm by quadrant

ID	ant. base	post. base	ant. apex	post. apex
1	33%	35%	14%	18%
2	39%	16%	16%	29%
3	40%	15%	22%	23%
4	61%	32%	6%	1%
5	35%	43%	19%	3%
6	60%	35%	3%	2%
7	22%	35%	32%	10%
8	30%	27%	25%	18%
9	23%	42%	8%	26%
10	61%	17%	5%	16%
11	38%	12%	17%	32%
12	52%	39%	5%	5%
13	50%	39%	5%	6%
14	63%	35%	0%	1%
15	74%	21%	2%	3%
mean	46%	30%	12%	13%
standard deviation	16%	11%	10%	11%
normality test:	Shapiro-Wilk test			
P value	0.47	0.07	0.17	0.07

This section shows the CT PTV3mm extension outside MR PTV5mm by quadrant

ID	ant. base	post. base	ant. apex	post. apex
1	3%	28%	40%	29%
2	0%	74%	21%	4%
3	0%	85%	3%	12%
4	0%	39%	45%	16%
5	1%	15%	35%	50%
6	10%	6%	31%	53%
7	15%	50%	2%	34%
8	5%	42%	36%	17%
9	15%	22%	42%	21%
10	4%	42%	30%	23%
11	0%	38%	60%	2%
12	22%	24%	22%	32%
13	5%	22%	35%	38%
14	13%	17%	37%	33%
15	4%	12%	48%	36%
mean	7%	34%	32%	27%
standard deviation	7%	22%	16%	15%
normality test:	Shapiro-Wilk test			
P value	0.02	0.11	0.34	0.92

Comparison of means of % volume outside MR PTV5mm vs CT PTV3mm

	delta ant. base	:a post. base	lta ant. apex	:a post. apex
mean	39%	-5%	-20%	-14%
standard deviation	18%	30%	22%	24%
normality test:	Shapiro-Wilk test			
P value	0.85	0.08	0.05	0.76
significance test:	two-tailed paired student's t-test* or signed rank test**			
P value	<0.001*	0.55*	0.008**	0.048*

Model parameters

	EUD a value	TCD50 [Gy]	gamma50
Rectum	-10	72	5

ref: AAPM TR166 and Radiother Oncol 2002;63:11e26

TCP for prostate CTV

					pairwise comparison 1			pairwise comparison 2		
**Patient ID**	**parameter**	**CTopt CT_CT**	**CTopt MR_C**	**MRopt MR_CTV**	**CTopt CT_CTV**	**MRIopt MRI_CTV**	**delta (CT-MRI)**	**CTopt MRI_CTV**	**MRIopt MRI_CTV**	**delta (CT-MRI)**
1	TCP [%]	91.1287	90.6685	90.5473	91.1287	90.5473	0.5814	90.6685	90.5473	0.1212
2	TCP [%]	90.8582	90.8753	90.452	90.8582	90.452	0.4062	90.8753	90.452	0.4233
3	TCP [%]	91.124	91.0111	90.7841	91.124	90.7841	0.3399	91.0111	90.7841	0.227
4	TCP [%]	90.92	86.5949	92.388	90.92	92.388	-1.468	86.5949	92.388	-5.7931
5	TCP [%]	91.0729	90.8408	90.4932	91.0729	90.4932	0.5797	90.8408	90.4932	0.3476
6	TCP [%]	92.295	92.1397	94.5725	92.295	94.5725	-2.2775	92.1397	94.5725	-2.4328
7	TCP [%]	93.3643	93.1808	94.8057	93.3643	94.8057	-1.4414	93.1808	94.8057	-1.6249
8	TCP [%]	91.1633	90.8103	91.6609	91.1633	91.6609	-0.4976	90.8103	91.6609	-0.8506
9	TCP [%]	91.2193	90.8035	92.2241	91.2193	92.2241	-1.0048	90.8035	92.2241	-1.4206
10	TCP [%]	91.0084	90.7845	91.9838	91.0084	91.9838	-0.9754	90.7845	91.9838	-1.1993
11	TCP [%]	91.4509	91.1497	92.7137	91.4509	92.7137	-1.2628	91.1497	92.7137	-1.564
12	TCP [%]	92.3345	91.8497	90.5404	92.3345	90.5404	1.7941	91.8497	90.5404	1.3093
13	TCP [%]	91.2498	91.2784	92.3599	91.2498	92.3599	-1.1101	91.2784	92.3599	-1.0815
14	TCP [%]	93.0811	92.9601	92.0198	93.0811	92.0198	1.0613	92.9601	92.0198	0.9403
15	TCP [%]	92.587	92.2965	90.9755	92.587	90.9755	1.6115	92.2965	90.9755	1.321
mean							-0.24423			-0.75181
median							-0.4976			-0.8506
standard deviation							1.23801			1.81553
interquartile range							1.8442			1.9873
normality test: Shapiro-Wilk test							p=0.4085			p=0.0323
significance test: two-tailed paired student's t-test* or signed rank test**							p=0.4575*			p=0.1688**

Model parameters

	EUD *a* value	TD50 [Gy]	gamma50
Rectum	8	76.9	4 and 8

ref: AAPM TR166 and Int J Radiat Oncol Biol Phys 2010;76(3 Suppl):S123e9

NTCP for rectum (Rx to prostate only and Rx to prostate + 1 cm SV)

Patient ID	parameter	CT	MRI	delta (CT-MRI)
1	NTCP [%] y5	0.0215468	0.143707	-0.12216
2	NTCP [%] y5	0.0221564	0.0474333	-0.02528
3	NTCP [%] y5	0.0755005	0.0660177	0.00948
4	NTCP [%] y5	0.278216	0.115152	0.16306
5	NTCP [%] y5	0.074617	0.0205132	0.0541
6	NTCP [%] y5	0.0475116	0.122526	-0.07501
7	NTCP [%] y5	0.522454	0.246214	0.27624
8	NTCP [%] y5	0.0401891	0.0349138	0.00528
9	NTCP [%] y5	0.177199	0.132911	0.04429
10	NTCP [%] y5	0.153662	0.0126198	0.14104
11	NTCP [%] y5	0.0749298	0.488852	-0.41392
12	NTCP [%] y5	0.148223	0.0293972	0.11883
13	NTCP [%] y5	0.141861	0.0403511	0.10151
14	NTCP [%] y5	0.320052	0.0454292	0.27462
15	NTCP [%] y5	0.186822	0.0443328	0.14249
mean				0.0463
standard deviation				0.17078
normality test: Shapiro-Wilk test				p=0.1154
significance test: two-tailed paired student's t-test				p=0.3114

Patient ID	parameter	CT (SV)	MRI (SV)	delta (CT-MRI)
1	NTCP [%] y5	0.0499451	0.265035	-0.21509
2	NTCP [%] y5	0.113635	0.087425	0.02621
3	NTCP [%] y5	0.154709	0.119142	0.03557
4	NTCP [%] y5	0.547285	0.162222	0.38506
5	NTCP [%] y5	0.121023	0.0869297	0.03409
6	NTCP [%] y5	0.121523	0.578005	-0.45648
7	NTCP [%] y5	0.980051	0.679045	0.30101
8	NTCP [%] y5	0.063001	0.0760028	-0.013
9	NTCP [%] y5	0.588484	0.63897	-0.05049
10	NTCP [%] y5	0.213644	0.0353609	0.17828
11	NTCP [%] y5	0.141194	0.646128	-0.50493
12	NTCP [%] y5	0.484653	0.156629	0.32802
13	NTCP [%] y5	0.181307	0.131498	0.04981
14	NTCP [%] y5	0.60251	0.140795	0.46172
15	NTCP [%] y5	0.342394	0.100389	0.24201
mean				0.05345
standard deviation				0.28348
normality test: Shapiro-Wilk test				p=0.3344
significance test: two-tailed paired student's t-test				p=0.4473

Patient ID	parameter	CT	MRI	delta (CT-MRI)
1	NTCP [%] y5	1.4468	3.65495	-2.20815
2	NTCP [%] y5	1.46683	2.13199	-0.66516
3	NTCP [%] y5	2.67524	2.50583	0.16941
4	NTCP [%] y5	5.01698	3.28387	1.73311
5	NTCP [%] y5	2.65994	1.41216	1.24778
6	NTCP [%] y5	2.13371	3.38399	-1.25028
7	NTCP [%] y5	6.75734	4.73297	2.02437
8	NTCP [%] y5	1.96571	1.83456	0.13115
9	NTCP [%] y5	4.0429	3.51972	0.52318
10	NTCP [%] y5	3.77491	1.11097	2.66394
11	NTCP [%] y5	2.66537	6.54987	-3.8845
12	NTCP [%] y5	3.70989	1.6859	2.02399
13	NTCP [%] y5	3.63221	1.96959	1.66262
14	NTCP [%] y5	5.36253	2.0874	3.27513
15	NTCP [%] y5	4.14692	2.06256	2.08436
mean				0.63539
standard deviation				1.96253
normality test: Shapiro-Wilk test				p=0.2659
significance test: two-tailed paired student's t-test				p=0.2304

Patient ID	parameter	CT (SV)	MRI (SV)	delta (CT-MRI)
1	NTCP [%] y5	2.18652	4.90228	-2.71576
2	NTCP [%] y5	3.26284	2.87308	0.38976
3	NTCP [%] y5	3.78727	3.33846	0.44881
4	NTCP [%] y5	6.9059	3.87476	3.03114
5	NTCP [%] y5	3.36385	2.86515	0.4987
6	NTCP [%] y5	3.37057	7.08455	-3.71398
7	NTCP [%] y5	9.04843	7.63706	1.41137
8	NTCP [%] y5	2.44929	2.68389	-0.2346
9	NTCP [%] y5	7.14427	7.42388	-0.27961
10	NTCP [%] y5	4.42247	1.84606	2.57641
11	NTCP [%] y5	3.62396	7.4625	-3.83854
12	NTCP [%] y5	6.52339	3.80984	2.71355
13	NTCP [%] y5	4.08767	3.50159	0.58608
14	NTCP [%] y5	7.22326	3.61903	3.60423
15	NTCP [%] y5	5.53694	3.07262	2.46432
mean				0.46279
standard deviation				2.3605
normality test: Shapiro-Wilk test				p=0.1247
significance test: two-tailed paired student's t-test				p=0.4603

Patient #	1	2	3	4	5	6	7	8	9	10	11	12	13	14	15
<u>CTV volume</u>															
MR prostate	23.9	28.69	38.38	37.4	33.52	18.98	71.81	37.95	18.57	40.21	20.08	27.08	26.2	15.62	23.52
MR prostate+1cmSV	27.12	30.62	41.39	42.14	37.62	21.72	76.22	42.76	21.22	43.52	24.01	29.25	28.62	17.69	26.5
MR prostate/SV	29.83	37.86	47.27	46.82	43.73	23.19	83.17	43.42	24.13	45.32	29.27	36.86	30.49	21.3	29.46
CT prostate	36.97	29.91	41.27	59.62	50.5	30.96	86.09	51.48	32.84	53.88	27.1	50.94	48.88	45.19	43.88
CT prostate+1cmSV	40.49	39.27	46.29	65.39	56.71	34.84	92.83	57.4	38.78	60.06	32.62	55.25	50.85	52.41	48.09
CT prostate/SV	42.84	43.03	50.31	68.56	64.4	43.16	101.48	58.2	43.92	62.82	41.42	65.28	51.75	58.63	52.29
<u>CTV+5mm volume</u>															
MR prostate	57.88	66.81	81.34	79.29	73.85	47.48	133.12	81.94	48.3	86.41	50.17	62.93	61.65	43.96	56.51
MR prostate+1cmSV	61.86	71.45	87.76	90.48	87.27	56.79	145.75	96.25	58.2	96.86	59.72	74.88	71.22	51.4	65.2
MR prostate/SV	71.45	96.19	109.78	104.02	108.51	64.14	167	99.05	73.17	106.53	77.61	101.07	81.3	68.65	78.65
CT prostate	80.45	68.49	90.83	120.02	104.62	67.65	162.6	107.06	77.05	111.2	64.74	104.06	102.64	94.26	90.96
CT prostate+1cmSV	90.51	94.92	104.01	133.82	121.33	78.71	180.38	122.31	94.14	127.25	79.13	119.29	111.16	113.34	104.67
CT prostate/SV	99.3	104.56	115.59	143.81	143.88	104.17	203.77	124.73	114.15	136.56	105.33	145.78	114.73	133.56	122.66
<u>CTV+3mm volume</u>															
MR prostate	40.27	47.35	59.49	57.83	53.17	32.86	102.08	59.46	32.85	63.33	34.16	44.45	43.11	31.52	39.26
MR prostate+1cmSV	42.66	50.78	64.16	65.51	61.88	38.99	110.38	68.59	38.69	70.17	40.96	50.92	48.63	36.37	45.07
MR prostate/SV	48.76	67.06	77.39	74.27	75.79	43.2	124.14	70.24	47.36	75.54	52.34	67.25	54.05	46.94	52.91
CT prostate	58.6	48.34	64.73	89.02	77.68	49.15	123.95	78.95	54.61	82.44	45.06	77.33	75.49	69.74	67.31
CT prostate+1cmSV	65.54	65.9	73.72	98.73	89.38	56.83	135.63	89.71	65.64	93.86	54.79	86.77	80.4	82.57	76.09
CT prostate/SV	70.99	72.59	81.67	105.34	104.65	74.2	151.63	91.34	78.24	100.24	72.62	104.9	82.58	178.29	86.93
<u>MR PTV5mm - CT PTV3mm (i.e. how much and where does the MR PTV extend outside the CT PTV)</u>															
prostate MR PTV5mn	8.86	22.21	25.85	19.96	10.32	5	21.51	11.64	6.01	14.22	9.43	3.46	7.44	6.37	6.52
%outside	15%	33%	32%	25%	14%	11%	16%	14%	12%	16%	19%	5%	12%	14%	12%
%overlap	85%	67%	68%	75%	86%	89%	84%	86%	88%	84%	81%	95%	88%	86%	88%
ant. base	2.88	8.71	10.34	12.16	3.6	2.98	4.83	3.51	1.41	8.74	3.62	1.81	3.73	4.03	4.81
post. base	3.13	3.55	3.84	6.39	4.46	1.76	7.49	3.11	2.51	2.48	1.15	1.34	2.91	2.25	1.36
ant. apex	1.27	3.53	5.71	1.27	1.94	0.15	6.98	2.94	0.5	0.7	1.65	0.16	0.34	0.02	0.13
post. apex	1.58	6.42	5.96	0.14	0.31	0.11	2.21	2.08	1.58	2.31	3	0.16	0.46	0.07	0.22
%ant. base	33%	39%	40%	61%	35%	60%	22%	30%	23%	61%	38%	52%	50%	63%	74%
%post. base	35%	16%	15%	32%	43%	35%	35%	27%	42%	17%	12%	39%	39%	35%	21%
%ant. apex	14%	16%	22%	6%	19%	3%	32%	25%	8%	5%	17%	5%	5%	0%	2%
%post. apex	18%	29%	23%	1%	3%	2%	10%	18%	26%	16%	32%	5%	6%	1%	3%
prostate+1cmSV MR	10.21	22.26	29.51	21.3	13.74	7.96	24.91	15.85	8.03	15.45	11.06	6.42	11.47	10.05	9.36
prostate/SV MR PTV5	14.4	40.03	40.69	27.09	25.32	9.47	34.81	16.76	11.75	20.75	16.48	15.5	19.15	5.74	15.88
<u>CT PTV3mm - MR PTV5mm (i.e. how much and where does the CT PTV extend outside the MR PTV)</u>															
prostate CT PTV3mm	9.59	3.74	9.24	29.7	14.14	6.67	12.34	8.65	12.31	10.26	4.31	17.87	21.28	32.15	17.32
%outside	16%	8%	14%	33%	18%	14%	10%	11%	23%	12%	10%	23%	28%	46%	26%
%overlap	84%	92%	86%	67%	82%	86%	90%	89%	77%	88%	90%	77%	72%	54%	74%
ant. base	0.26	0	0.01	0.12	0.08	0.64	1.89	0.41	1.89	0.45	0	3.96	1.11	4.3	0.67
post. base	2.72	2.78	7.85	11.5	2.17	0.4	6.11	3.64	2.66	4.28	1.65	4.27	4.67	5.42	2.13
ant. apex	3.85	0.8	0.27	13.46	4.89	2.07	0.19	3.15	5.17	3.12	2.59	4	7.36	11.81	8.29
post. apex	2.76	0.16	1.12	4.62	7	3.56	4.16	1.44	2.6	2.41	0.08	5.64	8.15	10.62	6.24
%ant. base	3%	0%	0%	0%	1%	10%	15%	5%	15%	4%	0%	22%	5%	13%	4%
%post. base	28%	74%	85%	39%	15%	6%	50%	42%	22%	42%	38%	24%	22%	17%	12%
%ant. apex	40%	21%	3%	45%	35%	31%	2%	36%	42%	30%	60%	22%	35%	37%	48%
%post. apex	29%	4%	12%	16%	50%	53%	34%	17%	21%	23%	2%	32%	38%	33%	36%
prostate+1cmSV CT P	13.89	16.71	15.48	29.55	15.85	7.99	14.79	9.31	15.48	12.46	6.12	18.3	20.65	41.22	20.25
prostate/SV CT PTV3	13.95	16.43	12.58	28.4	21.47	19.53	19.45	9.05	16.82	14.45	11.49	19.34	20.42	115.38	24.16
